# Ultrasound-Assisted Encapsulation of Sacha Inchi (*Plukenetia volubilis* Linneo.) Oil in Alginate-Chitosan Nanoparticles

**DOI:** 10.3390/polym11081245

**Published:** 2019-07-27

**Authors:** Mariela Elgegren, Suyeon Kim, Diego Cordova, Carla Silva, Jennifer Noro, Artur Cavaco-Paulo, Javier Nakamatsu

**Affiliations:** 1 Department of Science, Chemistry Division, Pontificia Universidad Católica del Perú PUCP, Av. Universitaria 1801, Lima 32, Peru; 2 Department of Engineering, Pontificia Universidad Católica del Perú PUCP, Av. Universitaria 1801, Lima 32, Peru; 3 Centre of Biological Engineering, University of Minho, Campus De Gualtar, 4710-057 Braga, Portugal

**Keywords:** sacha inchi, nanoemulsion, tocopherols, antioxidant, Nile red, protein loading

## Abstract

Sacha inchi oil is rich in essential and non-essential fatty acids and other types of bioactive agents like tocopherols and polyphenolic compounds, which are very well-known antioxidants. In this study, the encapsulation of sacha inchi oil in alginate (AL) and chitosan (CS) nanoparticles was achieved with the assistance of high-intensity ultrasound. Nanoemulsion is the most effective delivery and high stability system for lipophilic bioactive agents. Chitosan and surfactant concentrations were varied to study their effect on particle formulations. Size, zeta-potential, polydispersity, and stability of particles were determined in time to optimize the preparation conditions. Sacha inchi oil encapsulated in AL-CS nanoparticles showed a higher loading efficiency and stability for short and long periods compared with other vegetable oils such as olive and soybean. Also, because of the types of tocopherols present in sacha inchi oil (γ- and δ-tocopherols), a much higher antioxidant activity (95% of radical reduction in 15 min) was found in comparison with nanocapsules with olive oil, which contain α-tocopherols. The particles showed high efficiency of protein loading at high concentration of bovine serum albumin (BSA) and a low rate of leaching profiles in various testing media like simulated gastric and intestinal fluids with/without enzymes, that is, pepsin 0.1% (*w*/*v*) and pancreatin 0.1% (*w*/*v*), respectively.

## 1. Introduction

Sacha inchi (*Plukenetia volubilis* L.), also known as Inca peanut, is a native plant found in the Amazon rainforest of Peru, Ecuador, Brazil and other parts of South America [1,2,3,4,5]. The seeds of sacha inchi are characterized as rich in oil and proteins with aminoacids such as cysteine, tyrosine, threonine, and tryptophan [1,2,6]. Sacha inchi oil contains important macronutrients; bioactive compounds; heat-labile substances; and an unusually high content of essential fatty acids, that is, C18:3 omega-3 (α-Ln, cis, cis,cis-9,12,15-Octadecatrienoic acid; α-linolenic) and C18:2 omega-6 (L, cis,cis-9,12-octadecadienoic acid; α-linoleic) fatty acids, representing about 82% of the total oil content [5,6]. Besides the essential fatty acids, tocopherols, carotenes, phytosterols, and polyphenols are also present [5,6,7,8,9], these compounds are responsible for the health benefits and bioactivities attributed to sacha inchi. The beneficial effects of essential fatty acids on human health have been evaluated for treating diseases like arthritis, cancer, coronary heart disease, diabetes, hypertension, and inflammatory skin diseases [10,11,12]. Tocopherols and polyphenols are representative antioxidant agents commonly found in natural sources [11,12]. In the medical and pharmaceutical field, antioxidant activity, together with antimicrobial activity, is one of the most searched for bioactivities. Reactive oxygen species (ROS) are generally produced in the organism cells by oxidative stress and are closely associated with several diseases [13]. When ROS are formed during the pathogenesis of wounds and injuries, undesirable oxidative damage to proteins, nucleic acid, and lipids, as well as depletion of mitochondrial DNA from human skin, can occur [14]. 

Antioxidants prevent the damage caused by ROS through radical scavenging or prevention of the generation of these reactive species by iron binding [14], and thus they are important substances to protect the organism from the damage caused by the oxidative stress. Considering the nutrient composition of sacha inchi oil, it is an optimal candidate to become a natural antioxidant agent for medical and pharmaceutical applications.

High-intensity ultrasound has been extensively used over many years to synthesize nanostructured materials. Ultrasonic waves can produce high-energy chemical reactions in liquids in a brief time through acoustic cavitation, which provides a unique interaction of energy and matter [15,16]. Among many applications, the formulation of nanoemulsions (*w*/*o* or *o*/*w*) has been successfully carried out using ultrasound resulting in low polydispersity (homogeneous), size reduction, high stability, and no aggregation of particles [16,17,18]. In the present work, we employed ultrasound to form oil-in-water (*o*/*w*) nanoemulsions using sodium alginate (AL) and chitosan (CS) polymers to achieve stable nanostructured vehicles for medical and food applications. The sacha inchi essential oil was encapsulated to improve the bioactivity of AL-CS nanoparticles like antioxidant activity. Herein, we explore the encapsulation of other types of vegetable oils like soybean and olive oils, and their effects on the formulation and product properties of AL-CS nanoemulsions were studied and compared to sacha inchi oil. Emulsification is a very suitable system for encapsulating and delivering lipophilic components in aqueous media [19,20,21,22]. Among many polymeric nanocarriers for drug delivery system, CS and AL are the most broadly studied because of their biocompatibility, antimicrobial activity, non-toxicity, biodegradability, mucoadhesiveness, and longer in vivo circulation time, among others [21,22]. AL and CS are obtained from naturally occurring polysaccharides that form polyelectrolytes of opposite charges in solution. AL forms an anionic chain owing to the carboxylate groups, while CS forms a positively charged polymer chain owing to its protonated amine groups. Together, they can form stable polyelectrolyte complexes. Several factors must be considered for AL-CS polyelectrolyte complex formation, such as pH of the solution, molecular weight of polymers, and the ratio of CS to AL [22]. The effect of the type of oil and the chitosan and surfactant concentrations on the particle´s size, polydispersity, zeta-potential, and stability were evaluated. Encapsulation of sacha inchi in AL-CS nanoparticles was verified using fluorescence microscopy and Fourier-transformed infrared attenuated total reflectance spectroscopy (FTIR-ATR). Antioxidant activity of AL-CS nanoemulsions containing sacha inchi oil was also evaluated by measuring its free radical scavenging capacity.

## 2. Experimental

### 2.1. Materials

Commercial chitosan (CS) and sodium alginate (AL) obtained from Sigma-Aldrich (St. Louis, MO and Milwaukee, WI, USA, respectively), were used for the formulation of nanoparticles. Three different commercial vegetable oils were evaluated for encapsulation: sacha-inchi, olive, and soybean oils; all were produced in Peru and purchased from local suppliers (Inca-inchi, El Olivar, and Primor, respectively. Lima, Peru). Non-ionic surfactant poloxamer 407 (pluronic f127), lyophilized bovine serum albumin (BSA) with molecular weight 66 kDa, Nile red (Nile blue oxazone) dye, and other chemicals were purchased from Sigma-Aldrich and used without any further treatments.

### 2.2. Preparation of AL-CS Particles

Sacha inchi oil loaded AL-CS nanoemulsions were prepared according to methods reported by Lertsutthiwong et al (2009) and Natrajan et al. (2015), with some modifications [21,22]. 

**Figure 1:**
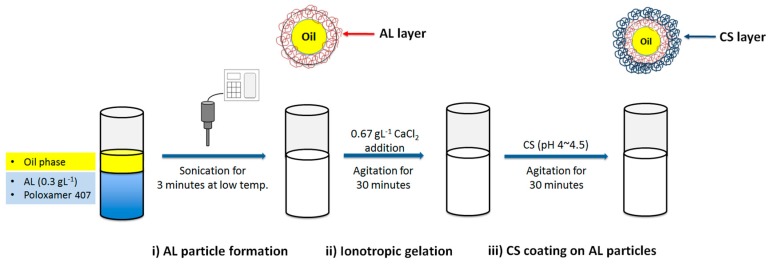
Experimental procedure for the formulation of sodium alginate (AL)-chitosan (CS) nanoemulsion encapsulating vegetable oils.

The AL-CS particles were prepared in three steps: (i) *o*/*w* nanoemulsion formation by ultrasound of a mixture of an AL aqueous solution, surfactant, and the organic phase (oil), (ii) ionotropic gelation of AL with calcium chloride by stirring for 30 min, and (iii) CS coating on AL particles by stirring for 30 min. In the first step (particle formation), high-intensity ultrasonication was applied to the mixture for 3 min with temperature controlled with an ice bath (7–10 °C). The ultrasonic probe was carefully positioned at the aqueous solution and oil interface. A 0.3 g·L^−1^ AL solution was prepared with distilled water and the pH was adjusted to 5–5.5. CS was dissolved in 1% acetic acid aqueous solution and pH was adjusted to 4.5–5 using NaOH solution. The experimental procedure is presented schematically in Figure 1.

In this study, we evaluated various factors that could affect the properties of the AL-CS particles, among them, the type of oil and the concentrations of CS and surfactant. For each case, the oil was added in the first step of the procedure and the volume ratio of AL solution to oil was kept constant at 9 to 1. 

Chemical structures of AL and CS were characterized by Proton nuclear magnetic resonance (^1^H-NMR) with a Bruker Ascend 500 MHz spectrometer (Billerica, MA, USA). The molecular weight of chitosan was determined by gel permeation chromatography (GPC) from Viscotek (Houston, TX, USA) with a refractive index detector and pullulan standards. The molecular weight of AL was determined by capillary viscometry with an Ubbelohde 1C viscometer (K = 0.02871 mm^2^·s^−2^) from Technical Glass Products (St. Dover, NJ, USA). 

After the formation of the AL-CS nanoemulsions under different conditions, the characterization of the particles was carried out measuring their average size, polydispersity index (PDI), and zeta-potential.

#### 2.2.1. Types of Oil

Three different edible oils were evaluated: sacha inchi, olive, and soybean oils. All the oil samples were produced by Peruvian companies (Lima, Peru) and used as they were obtained from the local market. The concentrations of AL and CS solutions were 0.3 g·L^−1^ and 2.0 g·L^−1^, respectively. 

#### 2.2.2. Concentration of CS

The effect of the concentration of CS in solution on the particle property was studied. Chitosan was dissolved in a 1% (*v*/*v*) acetic acid solution and the pH was adjusted to 5 using NaOH solution. Different concentrations of CS in solution were used, namely, 0, 0.3, 0.6, 1.5, and 2.0 g·L^−1^.

#### 2.2.3. Concentration of Surfactant

The surfactant plays a very important role in the formation of emulsion particles. Poloxamer 407 was added to the AL aqueous solution so that concentrations of 0.1, 0.2, 0.3, 0.5, and 1% (*w*/*v*) were obtained. 

### 2.3. Emulsion Stability (Phase Separation)

AL-CS nanoemulsions prepared with the different oils and different concentration of surfactant were stored for six months, and their physical stability was evaluated by observing if phase separation occurred (creaming phenomenon). Creaming index was obtained from the ratio between the height of the cream layer (*H*c) and the total height of cream and serum layers (*H*s + *H*c = *H*e, the total height of emulsion) according to Equation (1). *H*c and *H*s values were measured directly from the vials without shaking them.

(1) $$\mathrm{Creaming}\;\mathrm{index}\;\left(\mathrm{CI}\right)\left(\%\right)=\frac{Hc}{Hs+Hc}\times 100$$

### 2.4. Size, PDI, and Zeta-Potential

A Zetasizer Nano ZS (Malvern Instruments Inc., Worcester, UK) was used to obtain the values of average size, PDI, and zeta potential. Samples were diluted with ultrapure water before measurements in a ratio of 95:5 (water/sample). All measurements were performed with at least three samples. 

### 2.5. Fourier-Transform Infrared Attenuated Total Reflectance (FTIR-ATR) Spectroscopy Analysis

The encapsulation of sacha inchi oil in the AL-CS nanoemulsion was verified by FTIR-ATR spectroscopy using an FTIR/NIR (near infrared) frontier spectrophotometer (Perkin Elmer, Waltham, MA, USA). FTIR analysis is a simple and fast technique to determine the characteristic functional groups of samples. Besides that, it is not necessary to use any type of solvent to prepare samples for analysis. In the analysis of vegetable oils, a few drops of each oil sample were positioned in contact with ATR on a multi-bounce plate of crystal at controlled ambient temperature (25 °C). The lyophilized AL-CS nanoemulsion samples were also analyzed in the same manner. The ATR crystal was carefully and thoroughly cleaned using pure acetone after each run. IR spectra were recorded in the wavelength range of 4000 to 400 cm^−1^. The background spectrum of the empty ATR crystal was collected before proceeding to scan the sample.

### 2.6. Oil Encapsulation Efficiency Test Using Nile Red Dye

The oil was stained with Nile red, a lipophilic dye, before the preparation of AL-CS nanoemulsions. Then, 30 mL of AL-CS nanoemulsions prepared with stained vegetable oils were lyophilized. After lyophilization, the free unencapsulated oil was separated using ethanol. The lyophilized AL-CS nanoemulsions prepared with stained oil were washed with 20 mL of ethanol with stirring in a vortex for 1 min and then centrifuged. The supernatant ethanolic solution was used to determine the efficiency of oil encapsulation in the AL-CS nanoemulsions using UV-visible spectrophotometry at 553 nm, with maximum absorbance of Nile red dye. Dye concentration (DC) in the alcoholic solution was determined from a calibration curve at the same wavelength and was used to calculate the oil content in AL-CS nanoemulsion samples. The encapsulation efficiency was obtained from Equation (2). All measurements were performed using at least triplicate samples and the average value was determined.

(2) $$\mathrm{Encapsulation}\;\mathrm{efficiency}\;\left(\mathrm{EE}\right)\;\left(\%\right)=\frac{{\mathrm{DC}}_{\mathrm{initial}\;\mathrm{DC}}-{\mathrm{DC}}_{\mathrm{Free}\;\mathrm{DC}}}{{\mathrm{DC}}_{\mathrm{initial}}}\times 100$$

### 2.7. Evaluation of Antioxidant Activity 

The antioxidant activity was evaluated according to the 2,2’-azino-bis(3-ethylbenzothiazoline-6-sulphonic acid (ABTS) radical cation decoloration assay [23,24]. ABTS^•+^ free radical cation was obtained by the reaction between 7 mM ABTS solution and 2.45 mM potassium persulfate. The solution was stored for 12 h in the dark, at room temperature before use.

A total of 1 mL of the AL-CS emulsion was lyophilized before antioxidant evaluation. The ABTS^•+^ free radical solution was diluted with ethanol to reach an absorbance of 0.700 ± 0.025 at 734 nm and the absorbance of 3 mL of this solution was set as a control (*A*_control_). The lyophilized sample was added to 3 mL of the diluted ABTS^•+^ free radical solution and the absorbance was measured during one hour (*A*_sample_). All measurements were performed using at least triplicate samples and the sample’s free radical scavenging capacity was calculated with the following Equation (3):

(3) $$\mathrm{Radical}\;\mathrm{scavenging}\;\mathrm{effect}\;\left(\%\right)=\frac{A_{\mathrm{control}}-A_{\mathrm{sample}}}{A_{\mathrm{control}}}\times 100$$

### 2.8. Protein Loading Efficiency

BSA was studied as a model protein molecule to evaluate the loading efficiency and releasing behaviors of AL-CS particles. Various concentrations of 0.01, 0.03, 0.1, 0.5, 0.8, and 1.0 g·L^−1^. BSA were dissolved in the solutions containing 0.3 g·L^−1^ of AL and 0.5% (*w*/*v*) of surfactant. After adding BSA, the mixture was subjected to ultrasonication, ionotropic gelation, and CS coating. The BSA loading capacity of AL-CS particles was quantitatively evaluated by measuring the unloaded free BSA after emulsion formation. The separation of unloaded BSA from emulsion was acquired using a Vivaspin 20 filters (cut-off 100 kDa) by centrifugation (Beckman Allegra X155, Indianapolis, IN, USA) at 3000 rpm for 30 min at 25 °C. The BioRed DC protein assay was used to measure the concentration of protein in the filtered solution separated from particles. The loading efficiency was obtained using the same Equation (2) from the oil encapsulation efficiency calculation. The BSA loaded AL-CS nanoemulsions were further tested. All measurements were performed using at least triplicate samples and the average value was determined.

### 2.9. In Vitro Release Behavior

The release response of BSA loaded AL-CS nanoemulsions was studied with various testing medium, that is, simulated gastric and intestinal fluids with/without enzymes, that is, pepsin 0.1% (*w*/*v*) and pancreatin 0.1% (*w*/*v*), respectively, were prepared as described according to the United States Pharmacopeia (USP) guidelines [25]. A total of 5.05 mL of the AL-CS nanoemulsions was taken and mixed with the same volume of testing medium and incubated at the physiological temperature of 37 °C under gentle agitation [26]. After a certain time, like 1, 4, 8, 24, and 72 h, 10 mL of incubated samples was taken and placed in a Vivaspin 20 (cut-off 100 kDa), and then centrifuged at 3000 rpm for 30 min to separate the released BSA from the nanoemulsion [27]. The concentration of released BSA from particles was measured using the BioRed DC protein assay.

## 3. Results and Discussion

As an ecological, environmentally benign, non-toxic, and cost-effective method for the synthesis of nanoparticles, Kumar and his colleagues have studied the potential use of the leaf extract or seed oil of sacha inchi to produce silver or gold nanoparticles, and nanocatalyst [1,2,3,4]. They used sacha inchi extracts or oils as a reducing agent for silver or gold compounds and obtained small size particles (<100 nm). In the present study, we applied sacha inchi oil to obtain bioactive nanoemulsions formulated with two natural polyelectrolytes, AL and CS, and the effects on the particle properties were compared with olive and soybean oils. 

**Figure 2:**
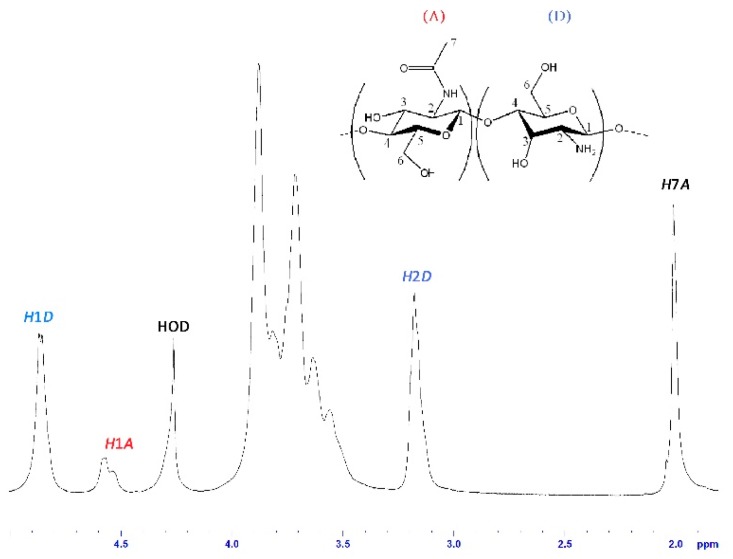
Proton nuclear magnetic resonance (^1^H–NMR) spectrum of chitosan (CS) solution in DCl/D2O, at 70 °C (500 MHz).

^1^H-NMR analysis of CS was obtained at 70 °C in DCl/D2O, with a Bruker Ascend 500 MHz spectrometer, with an AvanceIII HD console (Bruker, Billerica, MA, USA). A degree of deacetylation of 80.59% was calculated from the spectrum (Figure 2) based on the peaks for hydrogens in C1 (H1A, δ 4.54–4.58 ppm) and the acetyl group (H7A, δ 2.01 ppm) of the acetylated units and hydrogens in C1 (H1D, δ 4.84–4.86 ppm) and C2 (H2D, δ 3.15–3.17 ppm) of the deacetylated units [28]. A molecular weight (weight average) of 886 kDa was determined for CS with a Malvern Viscotek GPC system with an aqueous Novema Max analytical linear XL column and a refractive index detector. Pullulan standards (PSS Polymer Standards Service GmbH, 10–2000 kDa) were used as a reference and an HOAc/NaOAc buffer (pH = 4.5) was used as mobile phase [29].

For AL, 1H-NMR spectroscopy was used to determine a ratio of 1.57 mannuronic/guluronic units. This calculation is more complex [30] and is detailed in a previous work, in which the chemical structure of this AL sample was proven to be adequate for ionotropic gelation with calcium ions [31]. A molecular weight (viscosity) of 78 kDa was measured for AL by capillary viscosity in a 0.1 M NaCl aqueous solution at 25 °C using the Mark–Houwink equation with constants of δ = 0.92 and K = 0.0073 cm^3^/g [32].

### 3.1. Characterization of AL-CS Nanoemulsions 

#### 3.1.1. Types of Oil

**Figure 3:**
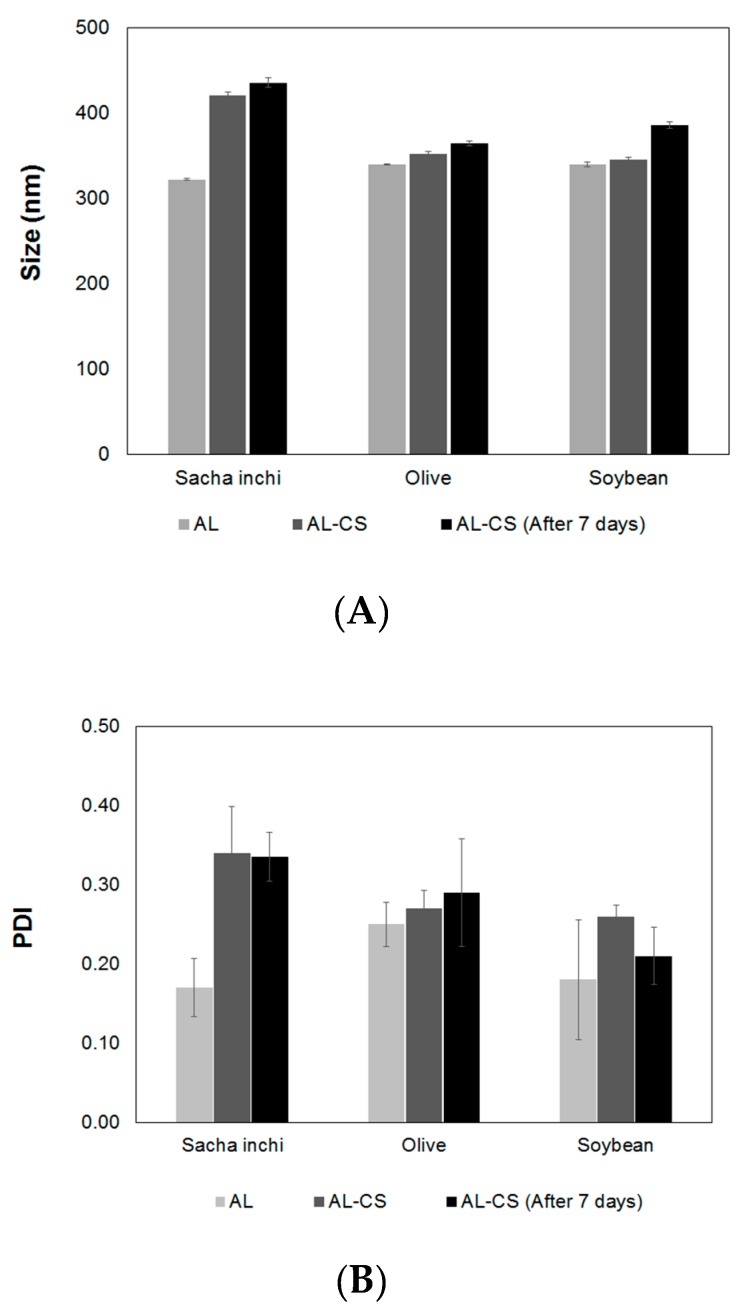

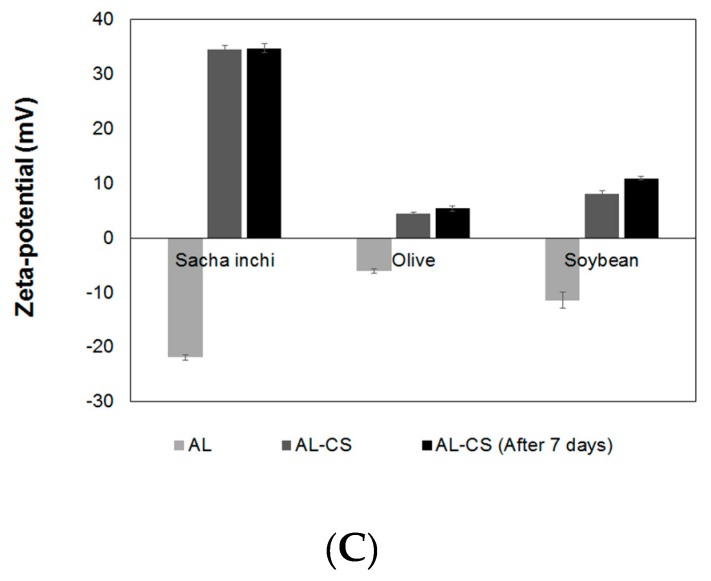
Effect of the type of oil on the properties of AL nanoemulsion (AL) and CS coated AL particles (AL-CS) on the particle size (**A**), size polydispersity (**B**), and zeta-potential (**C**), when prepared and after seven days. PDI, polydispersity index.

To determine the influence of the oil type on the properties of the nanoemulsion particles’ size, PDI and zeta potential were measured, and the results are presented in Figure 3. The oil in water AL nanoemulsion obtained in the first stage of the process was compared with the final AL-CS nanoemulsion. AL particles prepared with the different oils showed similar sizes with values around 320~340 nm, and an increase of particle size was observed after the last stage in which a coating with CS was formed (Figure 3A). Contrary to AL, CS is a high molecular weight cationic polyelectrolyte in solution; therefore, an increase in particle size is expected when a layer of CS deposits over the outer layer of AL as a result of electrostatic attraction [20]. 

In the case of particles prepared with sacha inchi oil, the size increase was much higher (30%) than for olive and soybean oils, 3% and 1%, respectively. After storage at ~5 °C for seven days, the particles were analyzed again. The size of particles prepared with sacha inchi and olive oils slightly increased, showing higher stability than the particles prepared with soybean oil. The size polydispersity of AL particles was quite low (≤0.25) and increased after CS coating (Figure 3B) in all samples. 

Figure 3C shows the changes of the zeta-potential values of oil loaded AL and AL-CS particles. The zeta-potential is an important parameter for the electrical characterization of nanoemulsions prepared with polyelectrolytes, as the adsorption of charged counter ions can be measured by its value and sign [33]. The type of oil used for the preparation of the AL emulsion greatly affected the zeta-potential. As expected, surface charges in the AL emulsions prepared with vegetable oils showed negative values because of the carboxylate groups of AL, a polyanionic chain [19,34]. The AL emulsion prepared with sacha inchi oil showed the most negative values (−22 mV), followed by soybean oil (−11 mV), and finally olive oil (−5 mV). After coating with CS, the surface charges of the particles changed from negative to positive as a result of the presence of the protonated amino groups in CS at a pH between 4.5 and 5 [22]. Among the three oils, sacha inchi oil presented the most dramatic change of its zeta-potential, from −22 to +35 mV, for AL and AL-CS particles, respectively. As mentioned before, the zeta-potential is an indicator of not only the surface charge of the particles, but also their stability [34]. CS-AL particles prepared with sacha inchi oil were expected to be very stable as their value of the zeta-potential was in the range of >|30|. This high stability could be the result of self-assembly of the two polymers around the oil core owing to their strong electrostatic attraction from their opposite charge nature [20].

#### 3.1.2. Concentration of CS Solutions

**Figure 4:**
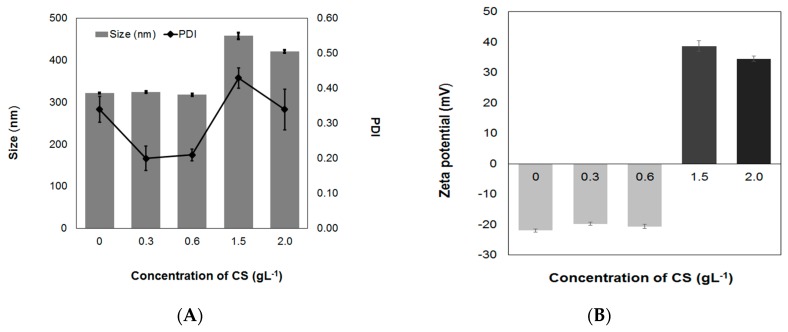
Effect of CS concentration on the properties of sacha inchi oil loaded CS-AL nanoemulsion. (**A**) Size/PDI and (**B**) zeta-potential.

The effect of the CS solution concentration on the formulation of AL-CS nanoemulsions was studied in terms of particles size, PDI, and zeta-potential. A low concentration of CS (≤0.6 g·L^−1^) resulted in no change in size nor zeta-potential of the AL-CS nanoemulsions. The zeta-potential remained with a negative sign, suggesting that the positively charged amino groups of CS were not enough to neutralize the negatively charged carboxylate groups in AL [35]. When CS concentration was increased to 1.5 g·L^−1^, the size increased and the zeta-potential became positive, as can be seen in Figure 4. This observation has been previously reported by Natrajan et al. (2015) and Choi et al. (2011) [22,33]. However, when the concentration of CS was higher than 1.5 g·L^−1^, the size and PDI of particles decreased and the zeta-potential was also reduced. Wang and his colleagues also observed this phenomenon with higher concentration of CS solutions, and reported that it may be the result of the saturation of ion logarithmic formed by electrostatic attraction forces between the –NH_3_^+^ of CS and the –COO^−^ of AL, as well as an increase viscosity of the CS solution, making the reaction difficult with the AL surface [35].

#### 3.1.3. Concentration of Surfactant

**Figure 5:**
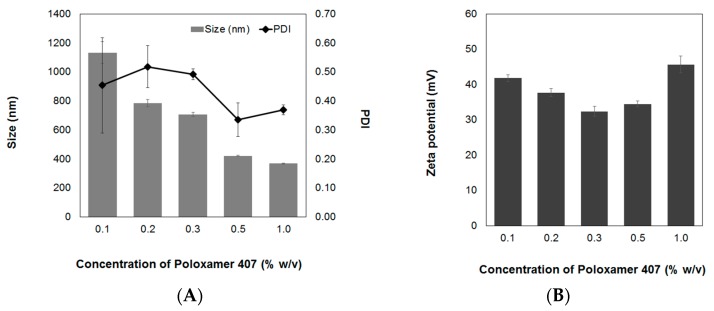
Effect of poloxamer 407 concentration on (**A**) size and PDI and (**B**) zeta-potential of sacha inchi oil loaded AL-CS nanoemulsion.

Surfactants are frequently used in the preparation of nanoparticles to optimize their physiochemical properties and stability. Different concentrations of the non-ionic surfactant, poloxamer 407, were used to encapsulate sacha inchi oil into the AL-CS particles. The effect on the particle size, PDI, and zeta-potential is evaluated as shown in Figure 5. Poloxamer 407 has a triblock structure containing a central hydrophobic block of polyoxypropylene (POP) and two identical lateral hydrophilic chains of polyoxyethylene (POE) [36]. Poloxamers have an amphiphilic character with both association and adsorption properties and improve solubilization and stabilization of compounds as a result of their notable physiological properties and low toxicity [37]. Poloxamers and poloxamines are non-ionic surfactants that have diverse applications in various biomedical fields such as drug delivery, medical imaging, as well as cancer therapy [38]. In a previous work, it was found that an increase of concentration of poloxamer 407 produces a reduction of both particle size and PDI [17]. Similar results were observed in AL-CS nanoemulsion encapsulating sacha inchi oil. According to the literature, non-ionic surfactants like poloxamer do not significantly affect the zeta-potential [39,40]. However, in our study, a variation of the zeta-potential was observed with poloxamer concentration (Figure 5B). The zeta-potential of sacha inchi oil encapsulated in AL-CS particles decreased when the poloxamer concentration changed from 0.1% to 0.3% *w*/*v* and increased when higher a concentration was used (0.5% and 1.0%, *w*/*v*).

### 3.2. Emulsion Stability

**Figure 6:**
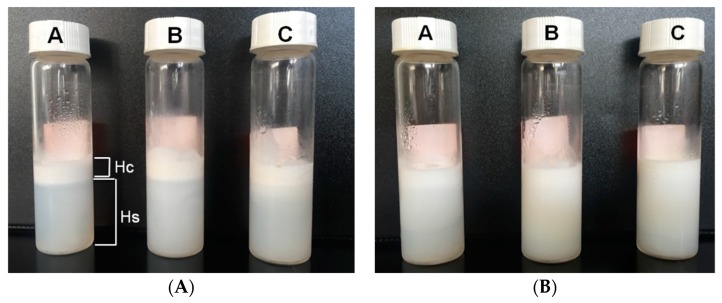
Phase separation images of AL-CS nanoemulsions (A: soybean oil, B: sacha inchi oil, C: olive oil) prepared with different surfactant concentrations, that is, (**A**) 0.1% and (**B**) 0.5%. The samples were stored for six months at 5 °C.

The phase separation of the AL-CS nanoemulsions for a long time (six months) was visually compared as an evaluation of their physical stability (Figure 6). Storage time, temperature, type of stabilizer, and type of oil phase often affect phase separation and show effects like creaming [41,42,43]. The phase separation of the serum from an emulsion often arises because of the flocculation and migration of oil molecules from smaller droplets to larger ones through the aqueous phase, which is known as the Ostwald ripening effect [41,44]. In AL-CS nanoemulsions, the concentration of surfactant and the type of oil affected the physical stability, producing different levels of serum separation.

In AL-CS nanoemulsions, poloxamer concentrations of 0.1% and 0.5% and the type of oil determined the phase separation of the system. When higher concentrations of poloxamer were used, better physical stability of AL-CS emulsion was observed, showing less phase separation than for lower poloxamer concentrations. Notable phase separation of serum with high turbidity was visually observed in samples prepared with 0.1% of poloxamer, showing a high rate of creaming (height of serum (*H*s) compared with the height of cream (*H*c)) [43,44]. In comparison with soybean and olive oils, AL-CS nanoemulsions encapsulating sacha inchi oil showed higher physical stability with a more stable emulsion. This observation is in accordance with the results from Figure 3. The zeta-potential, the direct indicator of particle stability, was the highest (+35 mV) in AL-CS nanoparticles encapsulating sacha inchi oil and remained after one week of storage.

### 3.3. FT-IR Analysis

**Figure 7:**
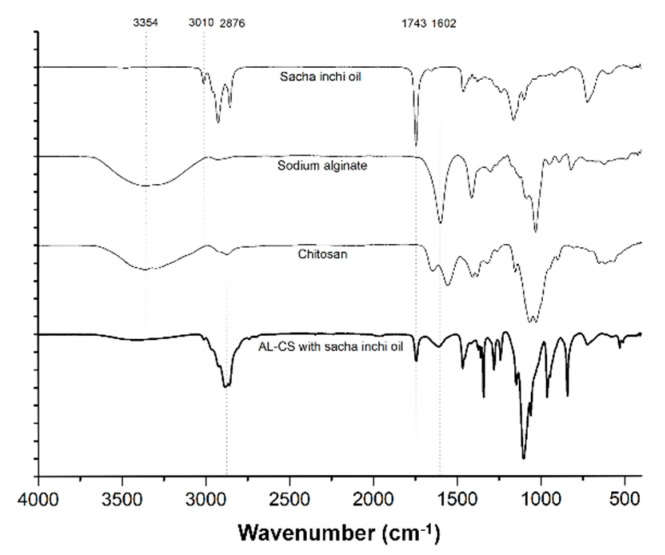
Fourier-transform infrared attenuated total reflectance (FTIR-ATR) of sacha inchi oil, AL, CS, and a sample of AL-CS nanoparticles with sacha inchi oil.

The encapsulation of sacha inchi oil in AL-CS nanoemulsions was verified by FTIR-ATR spectroscopy (presented in Figure 7). Spectra of fats and oil samples show peaks and shoulders that are attributed to their specific functional groups [45]. Gutiérrez et al. and Guillén et al. have previously reported the physicochemical characterization of sacha inchi oil using FTIR spectroscopy [46,47], which coincides with the spectra of our sample. The 3010 cm^−1^ band is attributed to the stretching vibration of *cis* olefinic C–H in carbon–carbon double bonds [46]. The two absorption bands at 2910 and 2854 cm^−1^ correspond to the methylene asymmetric and symmetric stretching vibration [47]. The characteristic band at 1743 cm^−1^ corresponds to the stretching vibration of the C=O group of triacylglycerols. The small band detected at 1650 cm^−1^ is assigned to the disubstituted *cis* C=C of the unsaturated acyl groups and the band at 1460 cm^−1^ is attributed to the bending vibrations of the CH_2_ and CH_3_ aliphatic groups [46,47]. The two bands at 1238 and 1163 cm^−1^ correspond to the stretching vibration of the C–O ester groups and to the bending vibration of the CH_2_ groups that are commonly found in many types of vegetable oils [47,48]. The band that appears at 730 cm^−1^ is attributable to the overlap of the methylene (CH_2_) rocking vibrations and to the out of plane vibrations of *cis*-disubstituted olefins [48]. These characteristic transmittance bands of sacha inchi oil were also detected in AL-CS nanoemulsions prepared with sacha inchi oil, which is evidence of the successful encapsulation of the oil into the nanoemulsion.

Besides the characteristic bands of sacha inchi oil in the nanoemulsion, the interaction between the amine groups of chitosan and the carboxylate groups of alginate can also be evidenced. The shift of the peak at 1660 to 1650 cm^−1^ is attributed to C=O stretching vibration and the disappearance of the –NH_2_ bending peak at 1602 cm^−1^ in chitosan was observed after the final stage of the AL-CS nanoemulsion formation [49]. The broad shoulder near 3400 cm^−1^ indicates the presence of hydroxyl and amino groups from AL and CS polymers.

### 3.4. Oil Encapsulation Efficiency 

**Figure 8:**
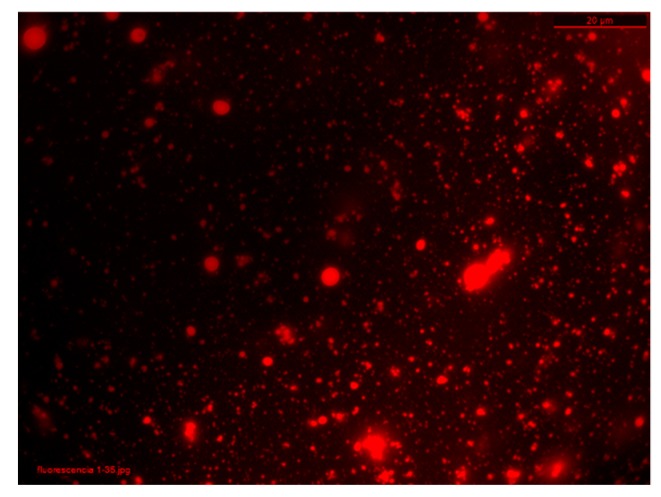
Microscopy images (x100) of AL-CS nanoemulsion prepared with sacha inchi: fluorescence filter TX2:BP 560/40 (red color corresponds to sacha inchi oil stained with Nile red).

Nile red is a lipophilic dye and has been used to stain lipids, cells, and oils [50,51,52,53]. In this study, Nile red was employed to stain vegetable oils to evaluate oil encapsulation efficiency. Figure 8 shows fluorescence microscopy images (Leica, Wetzlar, Germany) of a sacha inchi oil loaded AL-CS nanoemulsion, which is evidence of the successful loading of the oil. 

**Figure 9:**
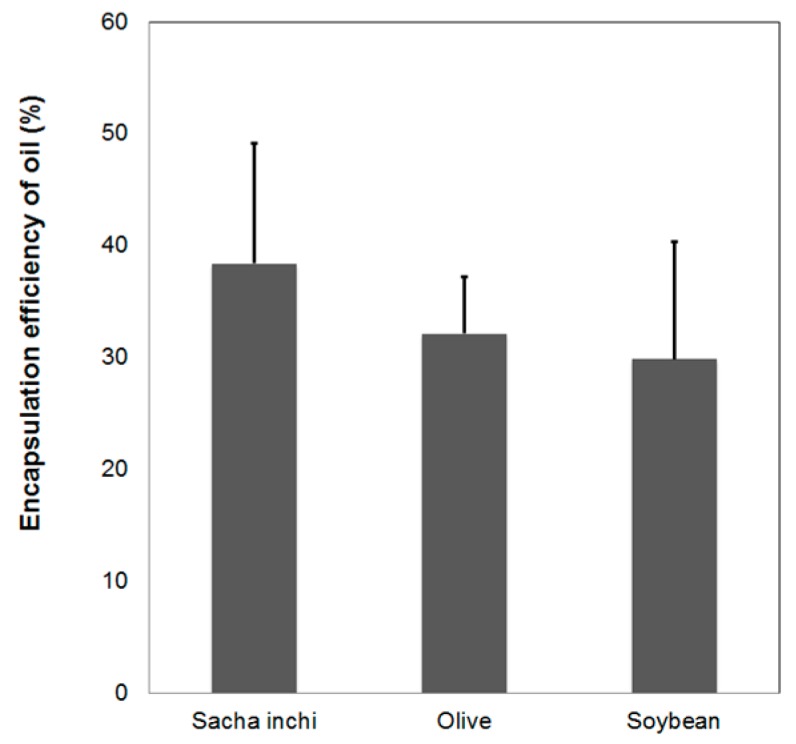
The oil encapsulation efficiency of AL-CS nanoemulsions prepared with different types of oil: sacha inchi, olive, and soybean (solution to oil ratio was fixed to 9:1).

The encapsulation efficiency was also determined and is presented in Figure 9. The unloaded oil from AL-CS nanoemulsions was separated by centrifugation using ethanol and the supernatant was analyzed with UV–Vis spectroscopy at 553 nm. Sacha inchi oil showed the highest value of loading efficiency compared with olive and soybean oils, although the total encapsulated oil quantity was less than 40% in the nanoemulsion. Encapsulation of olive and soybean oil in AL-CS nanoemulsions was lower, around 30~35%. This difference might be because of oil composition and physical properties such as density and viscosity. 

### 3.5. Evaluation of Antioxidant Activity

Most of the unrefined vegetable oils naturally contain antioxidant compounds that play an important role in protection against oxidation, both in oil stability and as nutritional benefits [7]. Sacha inchi seeds have recently become a popular nutrient source containing high levels of omega 3 fatty acids in the form of α-linolenic fatty acid (∼45%), various types of tocopherols (like α, β γ, and δ-tocopherol), phytosteroids, and phenolic compounds that are known antioxidant substances [5,7,8,9]. Among the many important desired bioactivities for medical and pharmaceutical applications, antioxidant activity is considered as one of the principal requirements. 

**Figure 10:**
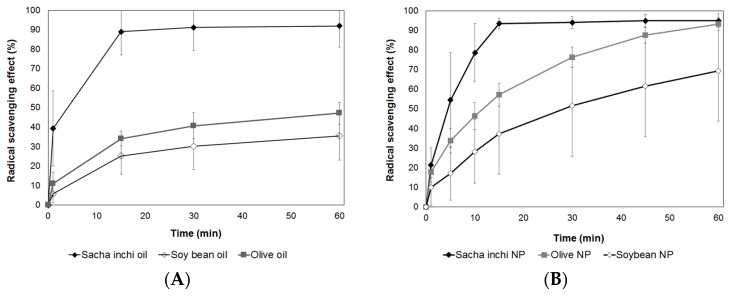
Evaluation of the antioxidant activity of (**A**) free vegetable oils (from sacha inchi, olive, and soybean) and (**B**) oil encapsulated in AL-CS nanoemulsions. *NP: Nanoparticles.

In this work, we evaluated the antioxidant activity of the three types of vegetable oil (from sacha inchi, olives, and soybeans) and the AL-CS nanoemulsions encapsulating the oils. The antioxidant activity of samples was measured for 60 minutes, and the cumulative percentage of inhibition (%) is presented in Figure 10A,B. Among the three types of oil, sacha inchi oil presented the highest and fastest capacity for radical scavenging. In 15 min, the reduction of free radicals reached near 90%. On the other hand, olive and soybean oil showed 35% and 25%, respectively, of free radical scavenging capacity in 15 min and reached up to 40% and 30%, respectively, of cumulative inhibition. Even though olive oil is well known to contain antioxidant components like omega fatty acids, tocopherols, phenolic compounds, as well as carotenoids similar to sacha inchi [54,55], the main difference between them is the type of the major tocopherols. Olive oil contains mostly α-tocopherol, while the main tocopherols in sacha inchi oil are γ- and δ-tocopherols, which are more effective antioxidants than α-tocopherol [56,57]. This explains that the radical scavenging capacity of sacha inchi oil was higher than that of olive oil. Moreover, compared with α-tocopherol, γ-tocopherol from sacha inchi is considered to be highly potent and very effective to trap reactive nitrogen oxides considered as toxic to the body [57]. The antioxidant activity of encapsulated vegetable oils in AL-CS nanoparticles was also evaluated. Chitosan is considered as a natural antioxidant polymer and its scavenging activity has been proven against 1,1-diphenyl-2-picrylhydrazyl radicals (DPPH•), hydroxyl radical (•OH), and superoxide radical (•O_2_^–^) [58,59,60]. The radical scavenging capacity of CS without oil incorporation reached up to 30% in 60 min. 

Encapsulated vegetable oils in AL-CS nanoparticles presented similar radical scavenging behaviors compared with their free oil samples (Figure 10B). Sacha inchi oil encapsulated in AL-CS nanoparticles showed the highest radical reduction rate in a short period, 95% in 15 min. As previously presented for the encapsulation efficiency of vegetable oils in AL-CS nanoemulsions (Figure 9), sacha inchi oil showed the highest value of encapsulation rate in AL-CS nanoemulsions and also possesses the strongest antioxidant capacity. The antioxidant activities of olive and soybean oils encapsulated in AL-CS nanoparticles gradually increased with time and reached 90% and 70% of radical scavenging, respectively, in 60 min. As the oils were encapsulated in stable particles, the release profile of oils from them might affect the gradually increasing antioxidant activity.

### 3.6. Protein Loading Efficiency

**Figure 11:**
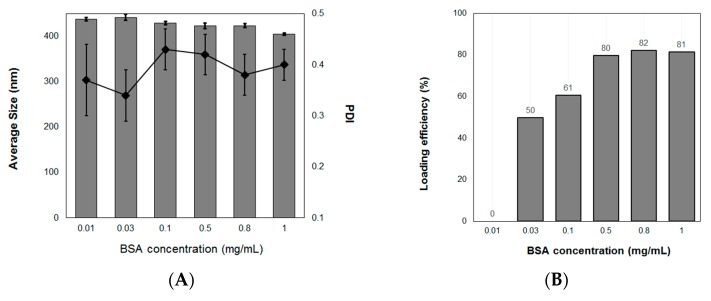
Effect of protein concentration on (**A**) the particle size and PDI and (**B**) loading efficiency of AL-CS nanoemulsion prepared with sacha inchi oil. BSA, bovine serum albumin.

The efficiency of protein loading in nanoparticles varies depending on the pH, formulation method (mechanical energy applied in the formulation), and ratio of polymers and protein. AL-CS nanoemulsions are positively charged because of the polycationic character of chitosan in acidic media, which is located on the outer layer of emulsion particles. Chitosan nanoparticles are often used as carrier materials to incorporate negatively charged macromolecules [61]. In this study, BSA was used to study the loading efficiency of sacha inchi oil loaded AL-CS nanoemulsions, because it has been broadly studied as a model of protein [62,63,64,65]. It has been reported that a higher concentration of protein produces larger particles and loading efficiency, because more interactions between the polymer and protein cause an increase in the particle size, which results in improving loading efficiency [17,64]. In our study, the size of AL-CS particles did not notably change, remaining in a range from 410 to 430 nm for all concentrations of BSA (Figure 11A). The loading efficiency of BSA increased with an increase in its concentration (Figure 11B). At the lowest concentration of BSA (0.01 g·L^−1^), the protein was not entrapped into the AL-CS particles. However, at concentrations higher than 0.5 g·L^−1^, the loading efficiency of the protein increased substantially to close to values near 80%, which remained with more BSA. In sacha inchi oil loaded AL-CS nanoemulsions, the protein might be positioned between the two oppositely charged polyelectrolytes, AL and CS, and thus the particle size did not change, because BSA is a small polymer (60 kDa) compared with CS or AL.

### 3.7. In Vitro Release 

**Figure 12:**
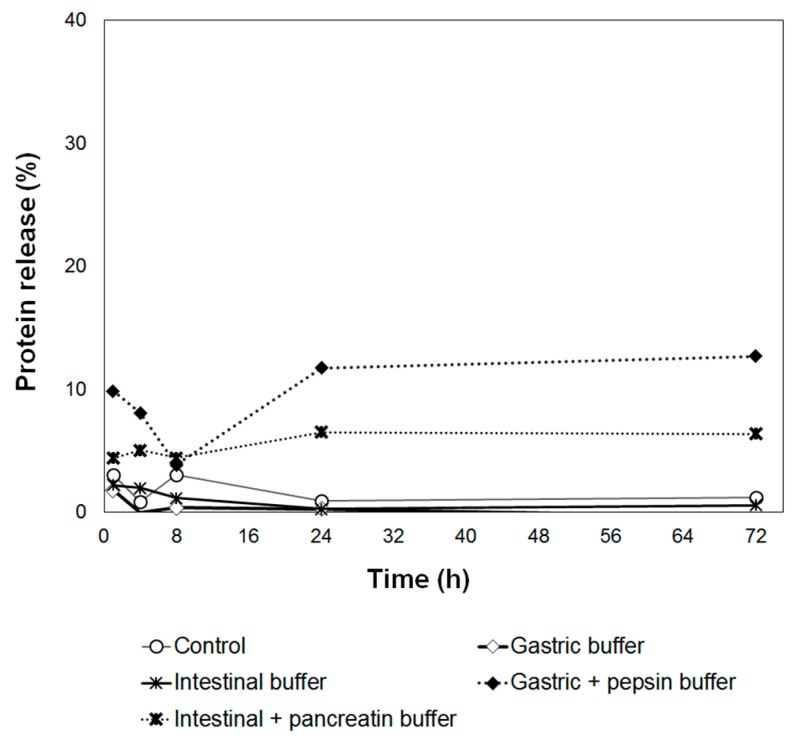
Cumulative release of BSA over 72 h in different mediums and the control sample.

After incubation of AL-CS nanoemulsion in various testing medium, the amount of BSA released from the particles was evaluated and is presented in Figure 12. In vitro protein releasing was monitored and a very low amount of protein was released during incubation. A slightly higher amount of protein was released in the digestive enzyme containing buffer compared with the non-digestive enzyme containing buffer, showing the positive effect of enzymes on the protein release from the particles. However, the maximum percentage of released protein only reached less than 12% (gastric buffer with pepsin enzyme) in 72 h.

In polymeric nanoparticles, the drug release rate depends on several factors: (i) drug solubility; (ii) desorption of the surface-bound or adsorbed drug; (iii) drug diffusion through the nanoparticle matrix; (iv) nanoparticle matrix erosion or degradation; and (v) the combination of erosion and diffusion processes [66]. Especially when the nanoparticle is coated by polymer, the release of the drug can be controlled by (iii) drug diffusion from the polymeric membrane. As the CS coating was performed over AL nanoparticles in the second process, the protein diffusion through CS membrane was the decisive factor on the release profile in AL-CS nanoparticles. The combination of AL and CS forms stable membranes as a result of entanglements and ionic interactions between the two polymers, which makes it hard for BSA to diffuse to the testing medium. Besides that, the interaction between BSA and each of the components in the particles might also contribute to its low release of BSA. As many proteins, BSA possesses amide groups and other polar functional groups. They can interact with CS, AL, or other compounds, either by ionic interactions, hydrogen bonds, or other van der Walls forces [66,67]. During the chain interaction in aqueous medium, BSA and CS form the non-covalently linked complexes without changing the conformation of BSA molecules. The BSA-CS complexes are potentially more functional and stable than the single polymer owing to the combination of the attributes of protein and CS polymer [68].

## 4. Conclusions

The ultrasound-assisted encapsulation of vegetable oils in AL-CS nanoparticles was successfully accomplished. Full characterization of AL-CS particles was carried out to optimize the physical properties and phase stability of particles. In the formulation of AL-CS nanoemulsions, the types of oils, concentrations of CS, and surfactant played decisive roles on the particle characteristics like size, polydispersity, and surface charge. The encapsulation of bio-active oils into AL-CS particles was evidenced by FT-IR and fluorescence microscopy analyses, and the sacha inchi oil showed a higher rate of encapsulation than other vegetable oils like soy and olive oils. Besides the oil encapsulation, the sacha inchi oil encapsulated AL CS particles presented the best behavior on the phase stability with a long period. Sacha inchi oil and AL-CS particles loading the same oil presented very strong antioxidant activity. Owing to the stable polymeric membrane and interaction with CS or other components, the release rate of BSA from AL-CS was very slow and only reached less than 12% (gastric buffer with pepsin enzyme) in 72 h. Sacha inchi oil encapsulated in AL-CS nanoemulsions displayed possible applications as a drug delivery system with high stability and controlled release properties.
